# Single-pulse phase-contrast imaging at free-electron lasers in the hard X-ray regime

**DOI:** 10.1107/S160057752001557X

**Published:** 2021-01-01

**Authors:** Johannes Hagemann, Malte Vassholz, Hannes Hoeppe, Markus Osterhoff, Juan M. Rosselló, Robert Mettin, Frank Seiboth, Andreas Schropp, Johannes Möller, Jörg Hallmann, Chan Kim, Markus Scholz, Ulrike Boesenberg, Robert Schaffer, Alexey Zozulya, Wei Lu, Roman Shayduk, Anders Madsen, Christian G. Schroer, Tim Salditt

**Affiliations:** a Deutsches Elektronen Synchrotron – DESY, Notkestraße 85, 22607 Hamburg, Germany; b Institute for X-ray Physics, University of Göttingen, Friedrich-Hund-Platz 1, 37077 Göttingen, Germany; c Third Institute of Physics, University of Göttingen, Friedrich-Hund-Platz 1, 37077 Göttingen, Germany; d European X-ray Free-Electron Laser Facility, Holzkoppel 4, 22869 Schenefeld, Germany; e Department Physik, Universität Hamburg, Luruper Chaussee 149, 22761 Hamburg, Germany

**Keywords:** phase problem, diffract-then-destroy, dynamical studies, pump–probe, sample delivery, XFELs, X-ray microscopy, phase contrast X-ray imaging

## Abstract

X-ray free-electron lasers (XFELs) have opened up unprecedented opportunities for time-resolved nano-scale imaging with X-rays. Near-field propagation-based imaging, and in particular near-field holography (NFH) in its high-resolution implementation in cone-beam geometry, can offer full-field views of a specimen’s dynamics captured by single XFEL pulses. To exploit this capability, for example in optical-pump/X-ray-probe imaging schemes, the stochastic nature of the self-amplified spontaneous emission pulses, *i.e.* the dynamics of the beam itself, presents a major challenge. In this work, a concept is presented to address the fluctuating illumination wavefronts by sampling the configuration space of SASE pulses before an actual recording, followed by a principal component analysis. This scheme is implemented at the MID (Materials Imaging and Dynamics) instrument of the European XFEL and time-resolved NFH is performed using aberration-corrected nano-focusing compound refractive lenses. Specifically, the dynamics of a micro-fluidic water-jet, which is commonly used as sample delivery system at XFELs, is imaged. The jet exhibits rich dynamics of droplet formation in the break-up regime. Moreover, pump–probe imaging is demonstrated using an infrared pulsed laser to induce cavitation and explosion of the jet.

## Abstract 2

XFEL radiation based on the stochastic SASE principle can be described by a low-dimensional configuration space. This space of pulse shapes can be sufficiently sampled before an imaging experiment. This approach is used to implement near-field holography of dynamic processes, demonstrated for the example of a micro-fluidic jet illuminated by the divergent wavefront emanating from a compound refractive lens nano-focus. Droplet formation in the break-up regime of the jet, as well as the hydrodynamic phenomena following plasma generation by an intense infrared laser pulse, can be imaged, based on flat-field corrected holograms and subsequent phase retrieval.

## 1. Introduction   

The capability to probe structural dynamics of a sample system is often an important prerequisite towards a more complete and quantitative understanding of physical processes. Experimentally, a major challenge is to cover the relevant time and length scales. This is in particular the case for complex fluids and soft matter where optical refraction, multiple scattering, hydrated environments, and opacity limit the application of electron and visible light pulses that are the most well established and versatile spatio-temporal probes at hand. With the advent of hard X-ray free-electron lasers (XFELs), imaging with high spatio-temporal resolution can now be combined with a large penetration power to probe the structural dynamics of liquids, complex fluids, and more generally soft and biological matter. While X-ray crystallography and X-ray spectroscopy cover molecular scales, coherent imaging with femtosecond X-ray pulses allows visualizing the structural dynamics over a wide range of length scales, from mesoscales to the nanometre range, as well as time scales from microseconds to sub-picoseconds. Importantly, sub-100 fs pulse illumination solves two major problems encountered in magnified X-ray imaging with synchrotron radiation. Firstly, the images are not blurred by the motion itself, not even by the smallest vibrations. Secondly, the radiation damage limit is overcome by the ‘diffract-before-destroy’ principle (Chapman *et al.*, 2014 ▸). Single-pulse imaging has also been demonstrated with white or pink beam synchrotron radiation, with a pulse length of the order of 10 ps to 100 ps (Luo *et al.*, 2012 ▸; Rack *et al.*, 2014 ▸; Olbinado *et al.*, 2017 ▸; Lee *et al.*, 2012 ▸). Temporal information can be inferred either from pump–probe schemes or for slower processes also by high-frame-rate acquisition. While the coherent flux of a single synchrotron pulse is still too small for many applications, which require nano-focusing and holographic recordings with geometric magnification, the high peak brilliance of XFEL pulses is ideally suited for ultrafast-high-resolution imaging.

Since the phase shifting part of the refractive index δ becomes orders of magnitude larger than the absorption coefficient β for hard X-rays and low-*Z* materials, much more information about the object is encoded in the phase shift rather than the absorption. From the multitude of phase-imaging methods available, only a few are compatible with single-pulse imaging at XFELs. The major constraint for single-pulse time-resolved imaging is the need for full-field capability without scanning of the object or any optical element. This constrains the transfer of some of the most powerful imaging methods developed for synchrotron radiation to XFELs, namely ptychography (Rodenburg *et al.*, 2007 ▸) on the nano scale; or correspondingly on meso- and macroscopic scales interferometric (Weitkamp *et al.*, 2005 ▸), and analyzer-based imaging methods (Chapman *et al.*, 1997 ▸). Ptychography with XFEL radiation has been demonstrated for static specimens (Schropp *et al.*, 2013 ▸; Sala *et al.*, 2020 ▸), and can be parallelized to some extent (Hirose *et al.*, 2020 ▸), but not yet with single-pulse capability. Coherent diffraction imaging (CDI) (Miao *et al.*, 1999 ▸), on the other hand, has been successfully implemented for single-pulse XFEL applications, including small biological particles such as bacteria or viruses (Seibert *et al.*, 2011 ▸; Neutze *et al.*, 2000 ▸; Sobolev *et al.*, 2019 ▸; Bogan *et al.*, 2008 ▸; Robinson *et al.*, 2015 ▸). It is, however, not suitable for larger specimens. Already eukaryotic cells (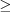
5 µm) are in most cases too large to fulfill the requirements of over-sampling the speckle pattern. A further limitation in the selection of phase-contrast techniques concerns the optical elements, which have to withstand the extreme conditions of XFEL pulses. While diffract-before-destroy can be implemented for samples delivered by replenishing systems as micro-fluidic jets, the concept obviously cannot be applied to the optics. Ease of implementation and compatibility with often demanding sample environments have to be considered as well. In this respect, both diamond-based Fresnel zone plates (David *et al.*, 2011 ▸) as well as beryllium compound refractive lenses (CRLs) (Lengeler *et al.*, 2005 ▸; Schroer *et al.*, 2005 ▸) offer clear advantages due to their radiation hardness. With all of the above limits and considerations in mind, propagation-based phase imaging (Nugent, 2011 ▸; Snigirev *et al.*, 1995 ▸; Cloetens *et al.*, 1999 ▸), and in particular its high-resolution implementation based on cone-beam geometry, also denoted as near-field holography (NFH), is particularly attractive.

NFH is by now well established at synchrotron radiation facilities (Mokso *et al.*, 2007 ▸) with dedicated end-stations (Salditt *et al.*, 2015 ▸; da Silva *et al.*, 2017 ▸). By using nano-focusing optics a secondary source of sub-100 nm size can be created. This source can be used to carry out magnified projection imaging in a cone-beam geometry. Due to the high degree of coherence of the X-rays, intensity contrast is generated by interference of the probing beam and the scattered waves, as first described by Gabor (1948 ▸). The cone-beam geometry allows full-field imaging with variable field of view and a maximum resolution down to the secondary source size (Davis *et al.*, 1995 ▸; Pogany *et al.*, 1997 ▸). Spatial resolution down to below 30 nm has been demonstrated (Bartels *et al.*, 2015 ▸; Khimchenko *et al.*, 2018 ▸). Several studies have already demonstrated projection and propagation imaging at XFELs (Rosenhahn *et al.*, 2009 ▸; Schropp *et al.*, 2012 ▸, 2015 ▸; Seiboth *et al.*, 2018 ▸; Vagovič *et al.*, 2019 ▸), but the intrinsic fluctuations of self-amplified spontaneous emission (SASE) illumination and the detrimental consequences for phase retrieval have not yet been solved. Previous methods presented for data treatment (Vagovič *et al.*, 2019 ▸) are not suited for the case of high-magnification imaging since these can induce a loss of small features in the data.

To illustrate this problem further, we consider the pre-processing step in holographic image processing. Before phase retrieval, the so-called standard flat-field correction is applied, written as (*r* − *d*)/(*e* − *d*), where *r* is the raw measurement, *i.e.* a hologram of the sample or event, *d* is the dark image, *i.e.* a readout of the detector without illumination, and *e* is the empty (flat-field) image, *i.e.* the measurement of the illumination without an object in the beam. This flat-field correction is in general only approximative, since it neglects the effects of free-space propagation (Homann *et al.*, 2015 ▸). This correction is necessary, however, in particular since nano-focusing of X-rays is often associated with strong inhomogeneities in the illumination. The errors introduced in the flat-field division decrease with smoother wavefronts. If this is not the case, empty beam division results in a loss of resolution, which – for cone-beam geometry – scales with the secondary source size (Homann *et al.*, 2015 ▸). Nevertheless, in order to separate contributions of the illuminating probe from the object, it is indispensable to perform the standard flat-field correction in holographic imaging as a necessary pre-processing step. Knowledge of the probe’s complex wavefield, obtained by a suitable characterization scheme (Robisch *et al.*, 2016 ▸; Hagemann & Salditt, 2017*a* ▸), would allow to take propagation effects into account and thereby reducing the artifacts. Note that alternative schemes devised to smoothen out for example the stripes associated with focusing by Kirkpatrick–Baez mirrors (Hubert *et al.*, 2018 ▸) require several exposures. In the context of single-pulse FEL imaging, this is not possible.

Due to the stochastic nature of the SASE process the wavefront fluctuates from pulse to pulse, with intensity and pointing (center-of-mass) of the beam being the most prominent examples. These variations make it practically impossible to record proper flat-field correction data, since the same pulse cannot be used *empty* and *filled*, unless a beam splitter (Li *et al.*, 2020 ▸) in front of the specimen was available to simultaneously record an empty image, *i.e.* of the same pulse, which is used for illumination of the object. For high magnification, and high FEL repetition rate, insertion of a beam-splitter between focus and object would be extremely challenging. In addition, it reduces the signal-to-noise ratio in the image.

In this work we aim to extend NFH from synchrotron to time-resolved XFEL imaging based on the following approach: a series of empty images *E* is recorded just before or after the data acquisition. This data series is analyzed by principal components analysis (PCA), and a suitable linear combination of PCA components is then used to decompose a given single-pulse hologram into contributions of object and illumination, *i.e.* to perform a *smart* flat-field correction. While this is similar to earlier work of Nieuwenhove *et al.* (2015 ▸) using synchrotron radiation, we here demonstrate that it is an enabling tool for NFH at XFELs. More precisely, we show that XFEL SASE pulses are well described by a low-dimensional configuration space. For the proof-of-concept we chose a well established sample delivery system for hydrated samples (soft and biological matter) at XFELs, namely a laminar micro-fluidic jet. We image the break-up instability of the jet, as well as strongly driven fluid dynamics following the generation of a plasma in the jet induced by a focused infrared (IR) laser pulse.

This manuscript is structured as follows: Section 2 details the experimental setup at the MID instrument of the European XFEL, including the nano-focusing optics. Section 3 explains the data pre-processing for holographic imaging with the fluctuating SASE illumination as a complicating factor for flat-field corrections and the subsequent phase-retrieval process. Section 4 presents imaging results for the micro-fluidic jet, driven (IR pump) and undriven (spontaneous break up). Finally, Section 5 concludes the work with a summary and outlook.

## 2. Experimental implementation at MID   

**Figure 1:**
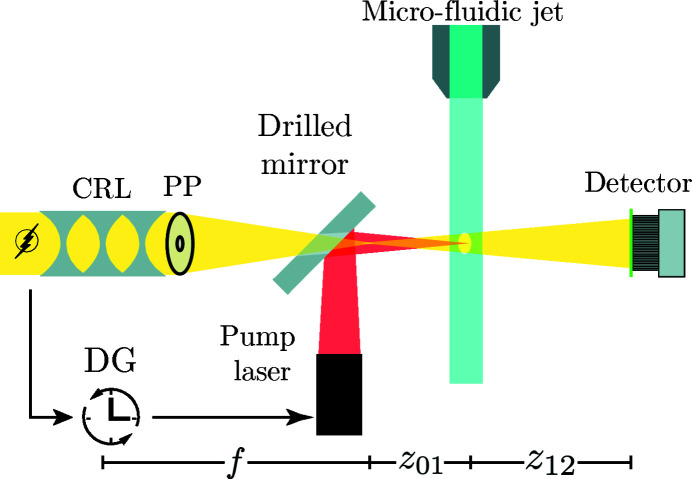
Sketch of the setup. The X-rays coming from the left have been generated in the SASE2 undulator and collimated by a CRL in the transport tunnel 729 m upstream to match the aperture of the nano-focusing CRL. A custom-made phase plate (PP) is used to correct beam aberrations (Fig. 3 ▸). The micro-fluidic jet is placed at a defocus distance of 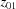 = 271.3 mm. The detector is placed at a distance of 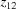 = 9670 mm behind the jet. A delay generator (DG) is used to trigger the pump laser according to the XFEL pulses. The laser is co-linearly coupled with the X-rays by the use of a mirror with a drilled hole for the X-rays to pass through.

The experiment has been carried out at MID (Madsen *et al.*, 2013 ▸; Tschentscher *et al.*, 2017 ▸) located at the European XFEL (Altarelli *et al.*, 2007 ▸; Decking *et al.*, 2020 ▸). Figure 1 ▸ shows a principal sketch of the experiment with focusing optics, the micro-fluidic jet and IR pump-laser. Since the lid of the multi-purpose sample chamber was removed, the setup was at ambient conditions.

**Figure 2:**
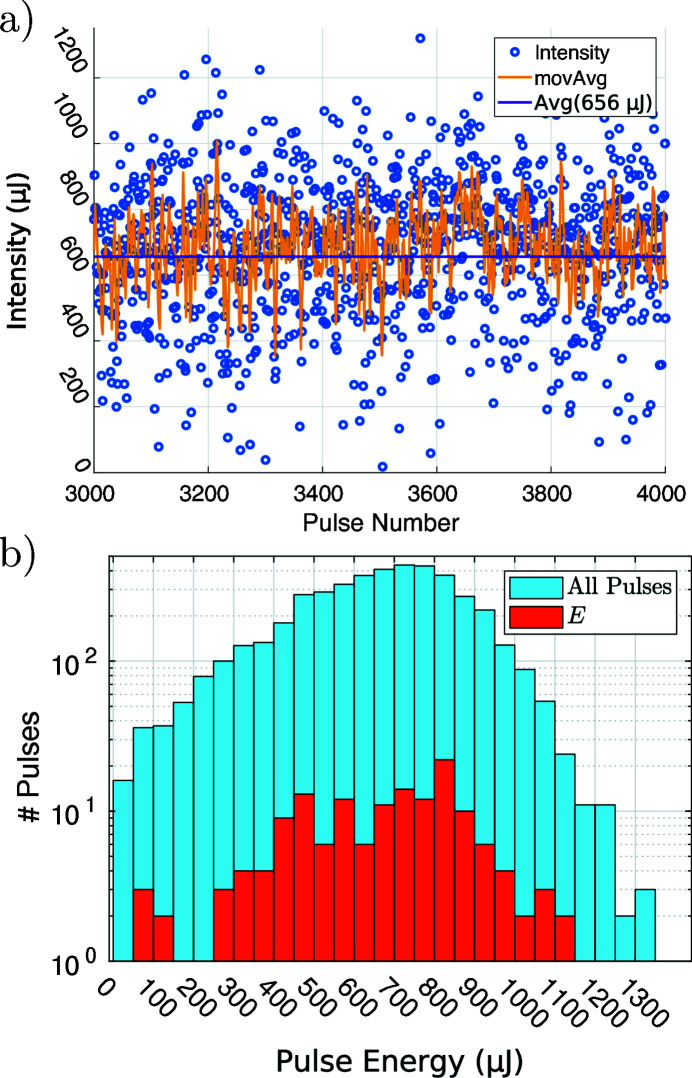
Pulse to pulse variations. (*a*) Time series of the energy per pulse for 1000 consecutive pulses of run 246. The moving average (movAvg) is taken over five adjacent pulses. The average pulse energy was calculated from the data of the whole run, consisting of 4000 pulses. (*b*) Histogram (blue) of pulse energies for the whole run. The fraction indicated in red stems only from empty pulses; these are used in Section 3.1 as input *E* for the PCA.

A photon energy of 17.8 keV was used at an average energy per pulse of 660 µJ. The filling pattern of electron pulse trains was set to 1 pulse per train, which results in a stroboscopic 10 Hz illumination and consequently data acquisition rate. It was the first experiment at EuXFEL carried out at this high photon energy and accelerator configuration in October 2019. Figure 2 ▸ shows the energy per X-ray pulse measured by an X-ray gas monitor located in the photon tunnel. Both the time series in Fig. 2 ▸(*a*) and the histogram in Fig. 2 ▸(*b*) show a considerable spread of the pulse energy for consecutive pulses and the complete pulse ensemble.

**Figure 3:**
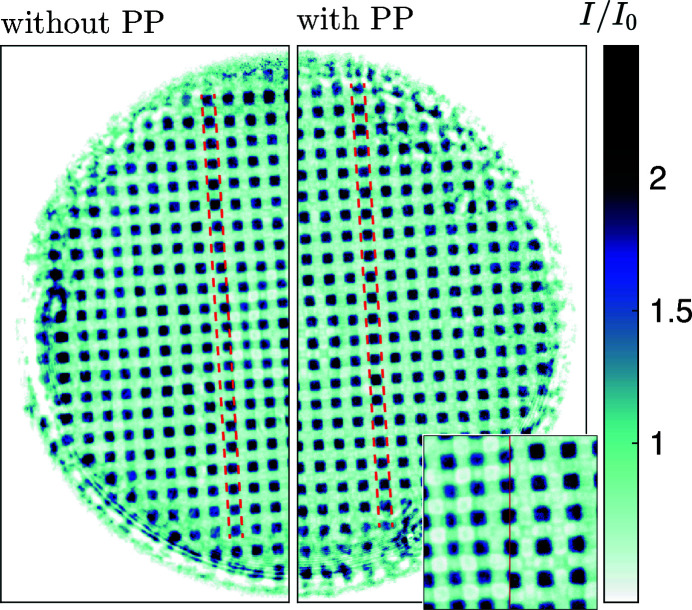
Effect of the corrective phase plate (PP). Flat-field corrected hologram of a gold mesh with 6 µm period and thickness of 37 µm. (Left) Hologram without PP. Strong distortions are visible, in particular in the center of the aperture. The dashed lines are provided as visual guides. (Right) Hologram with the PP inserted and aligned. The inset shows a zoom of the central region, indicating a significant improvement in contrast and edge steepness when the PP is used.

The X-rays were nano-focused by an aberration-corrected CRL (Seiboth *et al.*, 2017 ▸; Lengeler *et al.*, 2005 ▸) consisting of 50 beryllium lenses with an aperture of 300 µm and a radius of curvature of 50 µm, resulting in a diffraction-limited focus size of 94 nm (Schroer *et al.*, 2001 ▸). The aberration correction was achieved via a tailor-made 3D-printed polymer phase-plate (PP) (Seiboth *et al.*, 2020 ▸), which is placed at the exit of the CRL. The improvement of image quality is shown in Fig. 3 ▸. The image shows the flat-field corrected hologram of a gold grid close to a Talbot distance without (left) and with (right) the PP. On the left the distortions are visible as buckling of the lines of gold structures. Insertion of the PP turns these into straight lines. The inset also shows a significant improvement in contrast and edge steepness. The focal length of the nano-focusing CRL is *f* = 475 mm. Long-focal-length CRLs located upstream in the beamline were used to collimate the SASE pulses to a spot size of 1 mm at the entrance aperture of the nano-focusing CRL. This over-illumination was chosen to compensate for the pointing instability of the SASE process.

A micro-fluidic jet with a nozzle diameter of 40 µm was placed in a defocus distance of *z*_01_ = 271.3 mm and ran with a throughput of 20 mL min^−1^. The sCMOS detector (Andor Zyla 5.5 HF) is fiber-coupled with a 20 µm-thick LuAG:Ce scintillator for light conversion. It was placed *z*_02_ = 9942 mm behind the focus and the flight path between sample and detector was evacuated.

The jet was pumped by an infrared ns-laser (λ = 1064 nm, pulse energy 24 mJ) (Vassholz *et al.*, 2021 ▸). The laser was coupled co-linear with the X-ray beam and focused down to a spot size of 1.7 µm inside the water jet. The triggering and data acquisition was user-implemented and is detailed in another publication (Osterhoff *et al.*, 2021 ▸). The time resolution was limited by the pulse length of the laser of about 6 ns.

## 3. Data evaluation   

### 3.1. Flat-field synthesis for strongly fluctuating illumination wavefronts   

**Figure 4:**
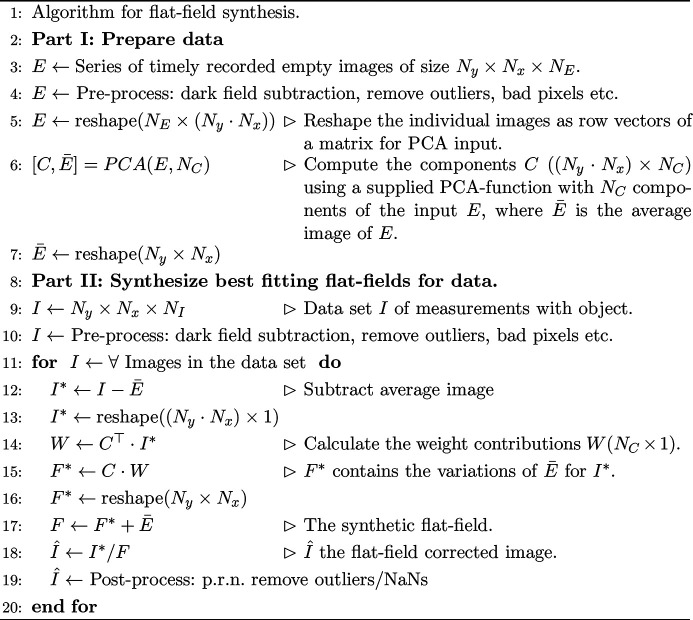
Algorithm for flat-field synthesis.

A series of *N*_*E*_ empty images *E*, each of dimension *N*_*y*_ × *N*_*x*_ pixels, is recorded just before or after the data acquisition with the same beam parameters. The best fitting empty image for each object hologram is computed from the PCA of *E*, following the ansatz shown for synchrotron radiation by Nieuwenhove *et al.* (2015 ▸). A pseudo-code algorithm for the flat-field synthesis is given in Fig. 4 ▸. The procedure can be easily implemented with the toolsets of Matlab or Python. The algorithm is basically separated in two parts. First (L. 2–7), the empty images *E* are assigned and the *N*_*C*_ components *C* are computed (L. 6). To this end we apply the PCA supplied by Matlab. Before the PCA is computed, standard operations like dark-field subtraction and removing bad detector pixels were applied. Also noise removal was applied to prevent the risk of noise over-fitting. To this end we used the removeOutliers-function provided by the *HoloTomoToolbox* (Lohse *et al.*, 2020*a* ▸) available here (Lohse *et al.*, 2020*b* ▸). Note that, while these pre-processing operations are not necessary, they help improve the result of the PCA.

Secondly, the *C* are used to calculate a synthetic flat-field *F*, which fits the data best (L. 8–20). The weights *W* are calculated (L. 14) by projecting the current image I* on the basis given by *C*. This calculation is straightforward compared with the minimization approach of Nieuwenhove *et al.* (2015 ▸). This flat-field synthesis has proven very robust for the current case of XFEL measurements but also for experiments at synchrotron radiation sources. The following section details our findings for the PCA analysis of the illumination during the experiment at MID. The analysis shown here was carried out on a specific run (a series of measurements) from the beam time, *i.e.* run 246 (Hagemann *et al.*, 2019 ▸). The empty images *E* were not sorted further for the following analysis, *e.g.* a criterion on pulse intensity was not applied. The images were used in the same sequence as they were recorded. In order to describe the variations in the data properly, *E* has to sample the possible configurations sufficiently. The red fraction of pulses in the histogram Fig. 2 ▸(*b*) indicates the number of pulses from a specific energy range, which are included in *E*. This shows that *E* samples most of the range of intensities. The following results serve as a typical example for the challenges encountered. Due to long-term drifts the ensemble of components *C* is not constant. Thus the *C* have to be evaluated anew for each run.

**Figure 5:**
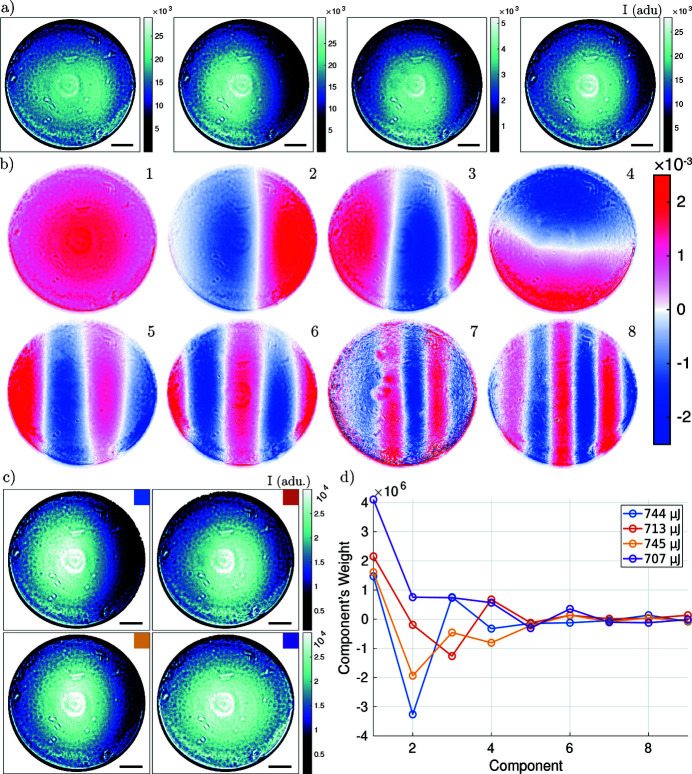
Flat-field synthesis. (*a*) Input data *E*; in total 150 empty images have been used as input. The selected empty images show the variation of intensity and movement of the illumination over the CRL’s aperture. (*b*) Components extracted by PCA. The strongest eight components out of 50 are shown. Each component has an ℓ2-norm of 1. (*c*) Empty beam images with approximately the same intensity. Only pulses from the bin with highest occurrence in the histogram Fig. 2 ▸ (700 µJ to 750 µJ) were selected. (*d*) The weights for the first nine components of the measurements shown in (*c*). Despite the similar FEL pulse energy, the measurements show some diversity in the mixing of the components. Color coding as in (*c*). The scale bar indicates 1 mm in the plane of the detector.

Fig. 5 ▸ summarizes the results of the PCA analysis for the nano-focused CRL illumination. An exemplary series of empty images is shown in Fig. 5 ▸(*a*), each image corresponding to an illumination with a single XFEL pulse. The four images illustrate the two effects mentioned earlier: (i) the non-constant pointing of the SASE beam can be recognized by the movement of the intensity distribution over the CRL aperture, and (ii) the intensity fluctuations are reflected by the different scaling of the colorbar, which are analyzed further in the time series of Fig. 2 ▸(*a*). The eight components with highest eigenvalue out of *N*_*C*_ = 50 are shown in Fig. 5 ▸(*b*). The full set of components is shown in the supporting information. These orthonormal components represent the variations from the mean of the input data. As we see for the SASE beam, the first component already corrects for the intensity variations between pulses. The subsequent components show pre­dominantly a variation in the horizontal direction reflecting a pointing instability along this axis. Higher components exhibit a continuous increase in the number of zero crossings, first and mostly in the horizontal but then also in the vertical direction. This is expected since the components are ordered with respect to the respective eigenvalues. Each component maximizes the variation of the data in a sub-space, which is orthonormal to the previous component. Since the pulse-to-pulse intensity fluctuation is large [see Fig. 2 ▸(*a*)], the average empty beam hologram does not well describe a particular acquisition, as further illustrated in the supporting information (Fig. S2). Thus, the first component for the SASE beam mainly corrects for the intensity variation between pulses, which in this case is the largest source of variance. Fig. 5 ▸(*c*) illustrates the situation of four pulses with similar intensities, the images have been identified on the basis of the intensity histogram of Fig. 2 ▸(*b*). The weights of the components describing these pulses are shown in Fig. 5 ▸(*d*). We conclude that intensity binning is not sufficient to classify the pulses. The weight distribution of the components shows clearly a different composition for each of the pulses. For this reason methods based on averaging multiple empty pulses for computation of the flat-field correction will fail.

**Figure 6:**
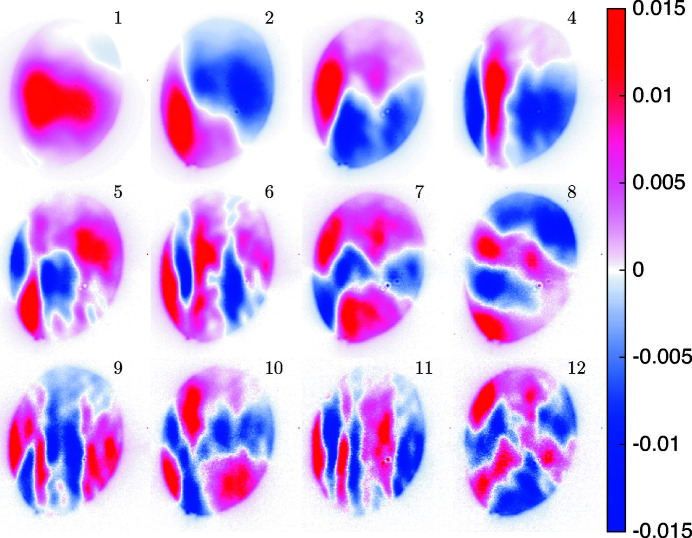
PCA components for the collimated SASE beam without nano-focusing CRL at MID. The 12 major components out of 60 are shown.

In order to image a larger field of view without geometric magnification, we used the unfocused but collimated SASE beam, which was also subject to a PCA. Fig. 6 ▸ shows the first 12 components out of *N*_*C*_ = 60 for the collimated beam, as calculated from 250 input images. The same flat-field synthesis as for the nano-focused CRL case was applied. The components show stronger structures than for the nano-focused beam; at the same time the variations per component are about a factor of ten higher. The oval shape of the illumination results from an aperture, formed by a hole in the mirror used for directing the infrared pump laser towards the micro-fluidic jet; see Fig. 1 ▸.

**Figure 7:**
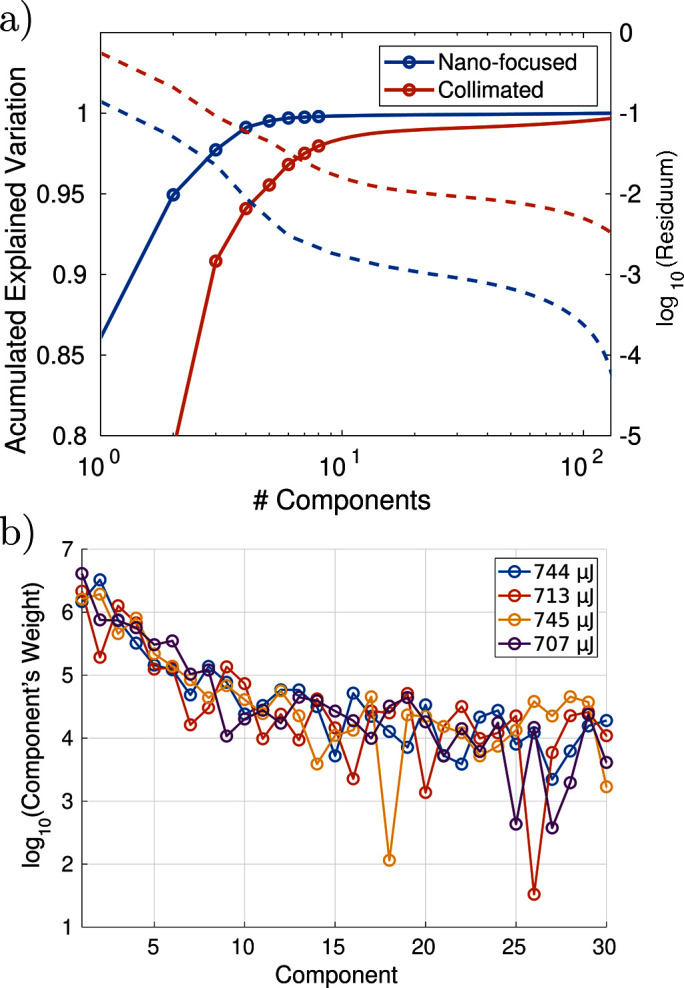
Additional flat-field analysis. (*a*) Comparison of the collimated and nano-focused SASE beam. The graph shows the explained variance (solid) and residuum (dashed) as a function of the number of components. (*b*) Decay of the modulus of the component weights on a logarithmic scale (nano-focus CRL), presented as an extension of Fig. 5 ▸(*d*). Even though the contribution drops well below the 1% level (relative to the weight of the first component), the higher components can contain localized information, which are beneficial for the correction.

Fig. 7 ▸(*a*) compares the explained variances, *i.e.* the described variability of the input data as a function of the number of components, for the nano-focused CRL and collimated beam case. The nano-focused CRL data are the same as in Fig. 5 ▸. The collimated beam data correspond to the components shown in Fig. 6 ▸. The solid lines indicate the accumulated explained variance of the input data-set *E* as a function of the number of components. Note that already for two components, 94.8% is reached in the nano-focused CRL case, compared with 79.1% for collimated beam. The dashed lines indicate the residuum of the data on a logarithmic scale. The residuum for the nano-focused CRL beam is an order of magnitude lower than for the collimated beam. This illustrates the fact that the nano-focus CRL acts as a filtering element by reducing the aperture. While this certainly allows for a better flat-field correction from less input data, the question arises in both cases of how to set a cut-off for the number of components. Fig. 7 ▸(*b*) extends the plot of Fig. 5 ▸(*d*) to 30 components, shown on a logarithmic scale. While a strong decay for the first ten components is observed, the decay levels off for the higher components. Since the trailing components encode local variations of the individual measurements, they cannot be neglected for a high-quality flat-field correction.

In the case of the nano-focused CRL beam, we found that a combination of 150 input images and 50 components resulted in a good compromise in terms of flat-field fidelity and numerical overhead.

### 3.2. Phase retrieval   

Next, we take advantage of the PCA flat-field correction scheme to perform phase retrieval (PR) on the corrected holograms. For PR we use the simple, but effective iterative algorithm of alternating projections (AP) (Luke *et al.*, 2002 ▸; Hagemann *et al.*, 2018 ▸). The physical constraints are coded/enforced in two projectors Π_M/S_ on the measurement M and sample S. A new iterate for the reconstructed specimen-function Ψ for the next iteration *n* + 1 of AP is given by 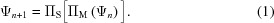
Note that instead of the conventional choice of amplitude and phase of the exit wave, we chose the representation of the specimen in terms of the projected index of refraction, *i.e.* the updated field is written as 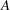 = 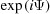 with 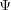 = 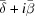. Here, 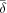 = 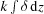 and 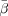 = 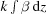 are the real and imaginary component of the index of refraction integrated over the thickness of the object, and *k* = 2π/λ is the wavenumber. The phase of the exit wave is hence simply 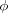 = 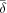 and the projected electron density can be written ρ_e_ = ϕ/(λ*r*_0_), where *r*_0_ is the Thompson scattering length. This reformulation involves alterations in Π_M_ but also allows a highly simplified implementation for some object plane constraints Π_S_ [see Wittwer *et al.* (2021 ▸) for details]. While the update scheme of the algorithm is parameter-free, the projectors Π_M/S_ require proper parameter settings reflecting the physical properties of the setup and the specimen. The projector Π_M_ onto the measurements 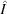 (magnitude constraint) is formulated such that it includes the propagation steps from the sample to the detector plane, and vice versa. For this purpose, the Fresnel free-space propagator 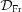 is used, 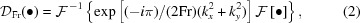
where *k*_*x*,*y*_ = 2*n*_*x*,*y*_/*N*_*x*,*y*_ are spatial frequencies in Fourier space with *n*_*x*,*y*_ ∈ [−*N*_*x*,*y*_/2…*N*_*x*,*y*_/2], *N*_*x*,*y*_ are the dimensions of the propagated array and Fr denotes the Fresnel number. In order to ensure proper sampling of the propagation kernel, the reconstruction array was padded to a size of 4096 × 4096 pixels. Since the propagation distance is not known and also aberrations in the beam and detector can slightly affect Fr, a first step is to calibrate the distance and correspondingly the effective Fr. The effective Fr is obtained by converting the coordinate system to an equivalent parallel beam geometry based on the Fresnel scaling theorem. The effective Fr is the standard Fr divided by the geometrical magnification *M* = *z*_01_/*z*_02_, yielding Fr_eff_ = Δ*x* ^2^/[λ(*z*_02_ − *z*_01_)*M*], with 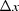 = 6.5 µm the pixel size of the detector and λ the X-ray wavelength. In the refinement of geometric parameters, we add a correction value *z*_cor_ to the measured value of *z*_01_ and recalculate the Fresnel scaling, within a certain search interval. With the Fr_eff_ from the interval, tentative reconstructions are computed, from which the sharpest is chosen by visual inspection. Once the best value for *z*_cor_ is found, the other parameters of the projectors are optimized. For this data the effective Fr_eff_ is 1.71 × 10^−3^ at an effective pixel size 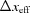 = 177 nm. The distance values reported in Section 2 are the corrected values. Note that the application of numerical gauges for the sharpness is problematic at this stage due to artifacts (notably stemming from the twin-image or flawed support). The PR is then carried out in a two-stage scheme: In the first stage a sample support is determined automatically, as will be described below. The second stage is started again from scratch, *i.e.* a matrix of zeros, but uses the support obtained in the first stage. For the first stage, 500 iterations are used, for the second 4000 iterations. These are the maximum values for the number of iterations, since the algorithms automatically tests if the remaining iterations can be skipped. For stage one, further iterations are skipped if the area of the support stays constant over the previous ten support adaptions. Stage 2 finishes if convergence, *i.e.* ||Ψ_*n*+1_ − Ψ_*n*_||_2_ < 10^−5^, is reached over the previous 100 iterations.

For Π_S_ the following constraints have been applied: support, ranges for 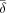, and enforcing a constant ratio 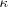 = 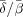. The details for the specific constraints are given in the following:

*Support.* For the water jet, which was positioned such that it did not fill the full field of view, the support mask was refined iteratively by using a combination of thresholding and morphological operations. The refinement starts in iteration 50 of stage 1. First, a threshold 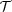 = 0.03 rad is applied,
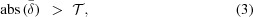
to define a rough estimate of the support. Next, an image erosion with a radius *r*_E_ = 3 pixels followed by a dilation with *r*_D_ = 5 pixels is applied. The erosion is used to remove small clusters of pixels after thresholding. Since larger structures are affected as well, the eroded contours have to be healed by a dilation. The value of *r*_D_ should be at least *r*_E_. However, if *r*_D_ − *r*_E_ becomes exceedingly large, the support diverges over subsequent iterations. For reasonable choices, this kind of support adaption was found to be very robust. This scheme is applied every five iterations. The values reported here for 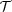, *r*_E_ and *r*_D_ are fairly typical and we expect that adaption to other specimens does not require significant changes. In order to suppress artifacts on the edge of the support in stage 2, the support has been smoothed by a Gaussian of 10 pixels full width at half-maximum.

*Ranges.* In general the range constraint can be used to limit the range of allowed values, which 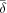 and 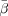 are known to vary. A common choice is the assumption of a *pure phase object*, *i.e.* the information is coded only in 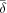, while 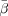 is forced to zero. We set the ranges for 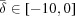.

κ*-ratio.* Since phase wrapping is not a matter of concern when updating the projected refractive index rather than the exit phase, the homogeneous object constraint can easily be applied also for specimens which exceed 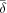 > 2π. Also this constraint is well justified since our sample consists only of water. The refractive index for water at 17.8 keV is *n* = 7.2 × 10^−7^ + *i*5.2 × 10^−10^ (Henke *et al.*, 1993 ▸). Therefore, the constant ratio 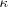 = 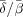 ≃ 1390 is enforced. The variation of this parameter in the range κ = 1300−1500 showed only a weak influence on the reconstruction’s quality. The advantage, however, to include this constraint is in an improved convergence of the algorithm.

A challenge for AP, but also for PR in general, is a faithful recovery of low spatial frequencies, *i.e.* the reconstruction of large homogeneous structures in the object. Note that the correct recovery of low spatial frequencies is of great importance, if the results are going to be analyzed in a quantitative manner. Small features are typically recovered correctly after a few iterations, while large structures need more iterations to converge. In order to speed up the convergence a Nesterov accelerated gradient (Nesterov, 1983 ▸; Ruder, 2016 ▸) has been implemented. The settings for the accelerator were η = 1.5 (step width) and γ = 0.85 (weight factor for the step). The acceleration has been applied in every iteration.

## 4. Results   

In the following we present the imaging results for the intrinsic (undriven) and laser-driven dynamics of the water jet with 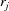 = 20 µm nozzle radius.

**Figure 8:**
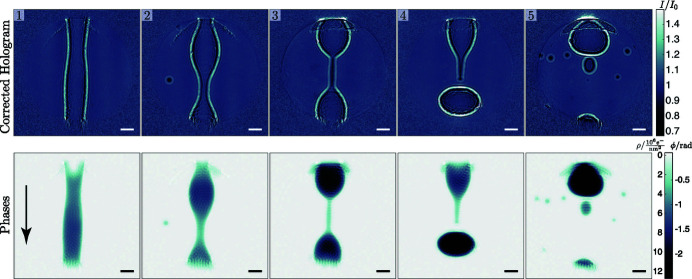
Single-pulse imaging of the intrinsic dynamics of the water jet in the break-up region. The upper row shows the flat-field corrected holograms and the lower row the corresponding reconstructed phase images. The two scales on the colorbar indicate retrieved phase in radians on the right and electron area density in 10^6^ e^−^ nm^−2^ on the left. The flow direction of the jet is indicated by the arrow in panel 1. The scale bar indicates 20 µm.

The water jet has two regimes: when leaving the nozzle, the jet is in a laminar flow, which destabilizes due to capillary forces and dynamical instabilities. The destabilization is followed by a break-up into smaller drops (Rayleigh, 1878 ▸). Fig. 8 ▸ shows a series of events in the break-up region of the jet. The jet throughput was set to 20 mL min^−1^, which results in a streaming velocity of *v*_*j*_ = 265.3 m s^−1^. The images were recorded approximately 6.3 mm under the nozzle, as can be deduced from the diameter of 31 µm of the jet in the first panel. The calculated break-up-length is 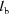 = 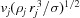 = 3.1 mm (Montanero & Ganan-Calvo, 2019 ▸), with ρ_*j*_ the density of water and the surface tension for water σ = 72.75 mN m^−1^. The series shows the formation of a drop in the break-up region. At first, the jet starts to undulate with increasing amplitude, as can be seen in panels 1 and 2; a video is provided in the supporting information. In panel 3 we observe a water filament of 6.3 µm diameter connecting two nodes. The fourth panel shows a similar filament shortly after ripping. In the last image the filament is dispersed in multiple sub-10 µm-diameter satellite droplets; the smallest droplet has a diameter of 4.2 µm. The volume of the large pinched off drops in panels 4 and 5 is approximately 66 × 10^3^ µm^3^, assuming rotational symmetry. This corresponds to spherical droplets with 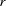 = 25 µm. From simple Rayleigh theory we would expect droplets with radius 29.4 µm and a volume of 106.5 × 10^3^ µm^3^ (Montanero & Ganan-Calvo, 2019 ▸). The structures at the top and bottom of the reconstructed phase images are artifacts. They appear at the positions where the jet enters and exits the illuminated region. For an illumination with Gaussian tails (not as sharp as for the CRL) we expect the artifacts to be less pronounced. In the following the results for the pumped dynamics are presented.

The setup for the study of the driven dynamics is similar to that reported by Stan *et al.* (2016*a* ▸), except that the role of pump and probe are exchanged. While the jet was pumped by a focused XFEL beam and probed optically in Stan *et al.* (2016*a* ▸), we here use a ns-IR laser to pump the jet, and probe it by XFEL pulses. In the defocus position, the XFEL beam is below the threshold of destruction, *i.e.* it has no visible influence on the jet dynamics, as verified by a ultra-fast optical camera. The interaction of short and focused optical/IR pulses with a water jet has been studied with advanced numerical schemes by Jeon *et al.* (2015 ▸), and differs from the case of ultra-short focused XFEL pulses (Paula *et al.*, 2019 ▸). Further, most previous studies (Lindinger *et al.*, 2004 ▸; Zhang *et al.*, 1987 ▸) with optical or IR pumping used femtosecond pulses, while in the current case an ns-pulse laser was used, which is expected to have a significant effect on the observed dynamics.

**Figure 9:**
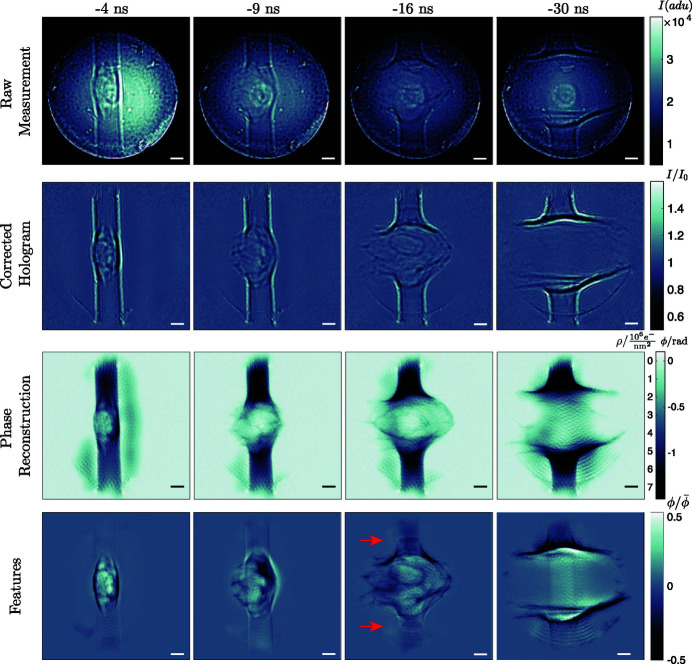
Pump–probe single-pulse imaging of an exploding water jet. From left to right the time delay is varied 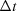 = −4 ns, −9 ns, −16 ns and −30 ns. Top to bottom illustrates the workflow of data processing. Starting with the raw hologram, the flat-field corrected hologram and the retrieved phases. The two scales on the colorbar of the reconstructions indicate retrieved phase in radians on the right and electron area density in 10^6^ e^−^ nm^−2^ on the left. The last row shows the reconstruction divided by the average phase. This highlights smaller structures present in individual images, which are otherwise hidden by large phase shifts. The red arrows in the −16 ns panel indicate shocks traveling along the jet in opposite directions. The scale bar indicates 20 µm.

The experiment with the pumping laser was performed in the laminar region of the jet. The average energy per IR pulse was 24 mJ. The time delays Δ*t* for the pump have been varied in the range from −30 ns to 5 ns in 35 steps. The delay is relative to the XFEL pulse, *i.e.* negative values indicate an IR pump before XFEL probe. For temporal and spatial overlap, and synchronization, we refer to the detailed procedures given by Osterhoff *et al.* (2021 ▸). For each delay a number of approximately 100 images was acquired. Fig. 9 ▸ shows the pumped dynamics of the jet for a selection of delays 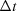 = −4 ns, −9 ns, −16 ns and −30 ns. A movie of all time delays is available in the supporting information. The figure shows the main analysis steps, as described in the previous sections, from top to bottom. The structural dynamics of the jet was extracted by averaging all reconstructions for a given time delay, and then dividing a particular reconstruction by the delay average to obtain the features shown in the last row. This representation allows to visualize small features and variations on top of large phase shifts. The smallest filaments visible in these feature maps have a diameter of 680 nm. For example at 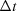 = −16 ns, we see above and below the explosion two ellipsoidal shapes, which are shock waves, propagating along the axis of the jet. This is in good agreement with Stan *et al.* (2016*a* ▸), who have observed the same effect optically. Note, however, that from the current (XFEL probe) data, one can in principle also quantify the corresponding density (Schropp *et al.*, 2015 ▸), and by application of the Tait equation (Hayward, 1967 ▸) also the pressure, which is not possible by optical detection. We further note that up to 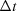 = −6 ns the shock front of the exploding water stays in a convex shape. Beyond that time point the small filaments separate and spread in the surrounding volume. For 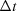 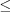 −9 ns another phenomenon is visible in the holograms. The shock wave in air is visible as well, but at a very low contrast of only 0.7%. This hindered a direct phase retrieval but still the shock velocity in air can be estimated to be in the range 7 km s^−1^ to 8 km s^−1^. Further visualizations for the in-air shock wave are presented in the supporting information.

## 5. Summary and outlook   

In summary, near-field holography offers unique advantages for time-resolved high-resolution imaging at XFEL sources, yielding single-pulse full-field imaging capability, combined with quantitative phase and amplitude contrast. Note that this need arises in time-resolved XFEL studies due to the fact that scanning-based coherent imaging and in particular ptychography, which is tremendously successful for synchrotron radiation, are not compatible with time scales faster than the microsecond range. However, up to now, near-field holography, known from synchrotron radiation, relied on a stationary illumination (probe), recorded pre- or post-hand of the actual image, in order to perform the required flat-field correction. The challenge of fluctuating SASE pulses has therefore been a major obstacle in transferring NFH from synchrotrons to XFELs.

In this work we have shown that a series of SASE pulses at EuXFEL, in particular when filtered by a nanofocusing X-ray optics, is well described by a low-dimensional configuration space, and that suitable representations of this space can be computed based on principal component analysis of a series of empty pulses. We could then compute the linear combination of PCA components best fitting the stochastic SASE pulse illuminating the object. This procedure resulted in high image quality of the corrected holograms and enables phase retrieval with minimal aberrations.

We have demonstrated this for two different classes of time-resolved imaging: fluid dynamics of a laminar jet spontaneously breaking up into droplets and the externally triggered (fluid) dynamics induced by an intense IR pulse focused onto the jet. Jetting, dropping and dripping exhibit a wealth of interesting hydrodynamic phenomena, which have been intensively studied by optical laser imaging, and are relevant for a range of technological applications. Due to higher spatial resolution and the quantitative electron density contrast, X-ray imaging can possibly yield important new insights in this field. However, in this study the selection of the water jet for the proof-of-concept experiment was primarily motivated by the fact that it can be used for sample delivery of hydrated objects, and is compatible with megahertz repetition rate due to the self-replenishment. Hence, for the same reason that jet systems have been successfully used in serial crystallography and CDI, they are also attractive delivery tools for full-field imaging. Obvious future extensions of this work could for example address the growth of particles in solution during a chemical reaction, or the delivery of fully hydrated biological particles, in particular bacteria or viruses.

While the typical length scales of the present example were primarily on the mesoscale, future extensions of the nano-focusing optics could scale the scheme down to well below 100 nm. For this purpose, X-ray waveguide filtering could offer a particularly high image quality and resolution down to about 30 nm (Bartels *et al.*, 2015 ▸). However, it is not clear yet to what extent (in particular with respect to higher pulse repetition rate) these optics can tolerate the high intensities present at XFELs. While the resolution can only be increased by the numerical aperture, hence by the nano-focusing optics, image quality and phase sensitivity are also important figures of merit. With highly performing phase retrieval schemes available, the current limits are still in the classical flat-field division itself (Homann *et al.*, 2015 ▸). In future this could be circumvented by an algorithmic solution similar to Hagemann & Salditt (2017*a* ▸), enhanced by the simultaneous reconstruction of modes (Hagemann & Salditt, 2017*b* ▸). In other words, rather than reconstructing intensity PCA components and dividing object intensities by (synthesized) flat-fields, the algorithm would fully work in the space of complex-valued coherent modes. Even with the current state of optics, this could significantly enhance the image quality and phase sensitivity. Further, the implementation of a second semi-transparent detector could be very beneficial for the robustness of the phase retrieval. This detector provides an additional simultaneous measurement at a different Fresnel number which can be used in the magnitude adaption step.

A particularly interesting class of problems, which can be studied by the NFH approach at XFELs, is the investigation of structural dynamics of pumped (driven) matter, in particular soft matter and complex fluids after optical or infrared excitation. The collective non-equilibrium dynamics and the relaxation back to equilibrium can be captured in a pump–probe scheme as illustrated here for the IR-pumped water jet, which showed interesting hydrodynamic effects related to recent experimental and numerical work (Stan *et al.*, 2016*b* ▸). With the same approach, after commissioning of the fs-laser system under installation at MID, much more extreme cases far from thermal equilibrium could be studied, as encountered in plasma physics or hot dense matter physics. Adding three-dimensional information in future experiments for these events is highly desirable. First approaches for multiple beam illumination have already been proposed (Villanueva-Perez *et al.*, 2018 ▸) but are not yet compatible with magnified NFH. An NFH compatible implementation could use splitting and bending X-ray waveguides, which provide a fan of clean illuminations with high divergence for magnified imaging.

To this end, a major limitation in exploiting the full potential of the EuXFEL’s capabilities for making movies of individual events at 4.5 MHz is in the lack of suitable detectors combining sufficient spatial resolution (small pixel size), dynamical range, and a tailored frame rate.

## Supplementary Material

Supporting Information on beam stability, phase retrieval and in-air shock-waves. DOI: 10.1107/S160057752001557X/il5056sup1.pdf

Pulse to pulse fluctuations of the SASE pulses focused by the Be-CRL stack. The color bar adapts for each frame. The scale bar indicates 1 mm. DOI: 10.1107/S160057752001557X/il5056sup2.mp4

The flat-field correction with increasing number of components used for computing the linear combination on a particular hologram. Not all divisions are shown, from NC = 10 to 20 every second and from N_C = 20 to 50 every fifth division result is shown. DOI: 10.1107/S160057752001557X/il5056sup3.mp4

Zoom on the ripple-like artifact. Same frames shown as in Fig. S3. DOI: 10.1107/S160057752001557X/il5056sup4.mp4

The intrinsic jet undulations shortly before break-up. DOI: 10.1107/S160057752001557X/il5056sup5.mp4

The temporal evolution of the exploding jet within 30 ns after the IR laser pulse has hit the jet. This figure is the animated version of Fig. 8 of the main text, showing the different processing stages of the data. DOI: 10.1107/S160057752001557X/il5056sup6.mp4
