# Supplementary material for: Single-pulse phase-contrast imaging at free-electron lasers in the hard X-ray regime
Source: J Synchrotron Radiat. 2021 Jan 1;28(Pt 1):52–63. doi: 10.1107/S160057752001557X (PMC7842230; doi:10.1107/S160057752001557X)
Supplement: Supplementary file 1 [file s-28-00052-sup1.pdf]

Supplemental: Single-Pulse Phase-contrast Imaging at Free-Electron Lasers in the hard X-ray Regime

JOHANNES HAGEMANN,[a]* MALTE VASSHOLZ,[b] HANNES HOEPPE,[b]
MARKUS OSTERHOFF,[b] JUAN M. ROSSELLÓ ,[c] ROBERT METTIN,[c]
FRANK SEIBOTH,[a] ANDREAS SCHROPP,[a] JOHANNES MÖLLER,[d] JÖRG HALLMANN,[d]
CHAN KIM,[d] MARKUS SCHOLZ,[d] ULRIKE BOESENBERG,[d] ROBERT SCHAFFER,[d]
ALEXEY ZOZULYA,[d] WEI LU,[d] ROMAN SHAYDUK,[d] ANDERS MADSEN,[d]
CHRISTIAN G. SCHROER[a,e] AND TIM SALDITT[b]

[a]Deutsches Elektronen Synchrotron – DESY, Notkestraße 85, 22607 Hamburg, Germany, [b]Institute for X-ray Physics, University of Göttingen, Friedrich-Hund-Platz 1, 37077 Göttingen, Germany, [c]Third Institute of Physics, University of Göttingen, Friedrich-Hund-Platz 1, 37077 Göttingen, Germany, [d]European X-ray Free-Electron Laser Facility, Holzkoppel 4, 22869 Schenefeld, Germany, and [e]Department Physik, Universität Hamburg, Luruper Chaussee 149, 22761 Hamburg, Germany. E-mail: johannes.hagemann@desy.de

x-ray near field imaging; **phase retrieval**; **free electron laser**; **fast processes**

Abstract

This supplement provides additional visualizations concerning the quality of the raw data, data pre-processing, influence of the number of components used for flat-field synthesis and movies of the jet phenomena.

1. Important Information

Read me

This PDF contains videos. For playback it is necessary to use the **Adobe Acrobat Reader**.

If the videos do not show, check for the ”3D content enable” dialog near the toolbar and scroll to the end document. This should enable the embedded videos.

Alternatively, on Windows **SumatraPDF** is able to extract the videos from the PDF and play them externally.

2. SASE Fluctuations

Figure S1 illustrates the fluctuations shown in Fig. 4(a) of the main text in an animation of 50 consecutive pulses. A circular mask has been applied to the images. White corresponds in this case to negligible intensity.

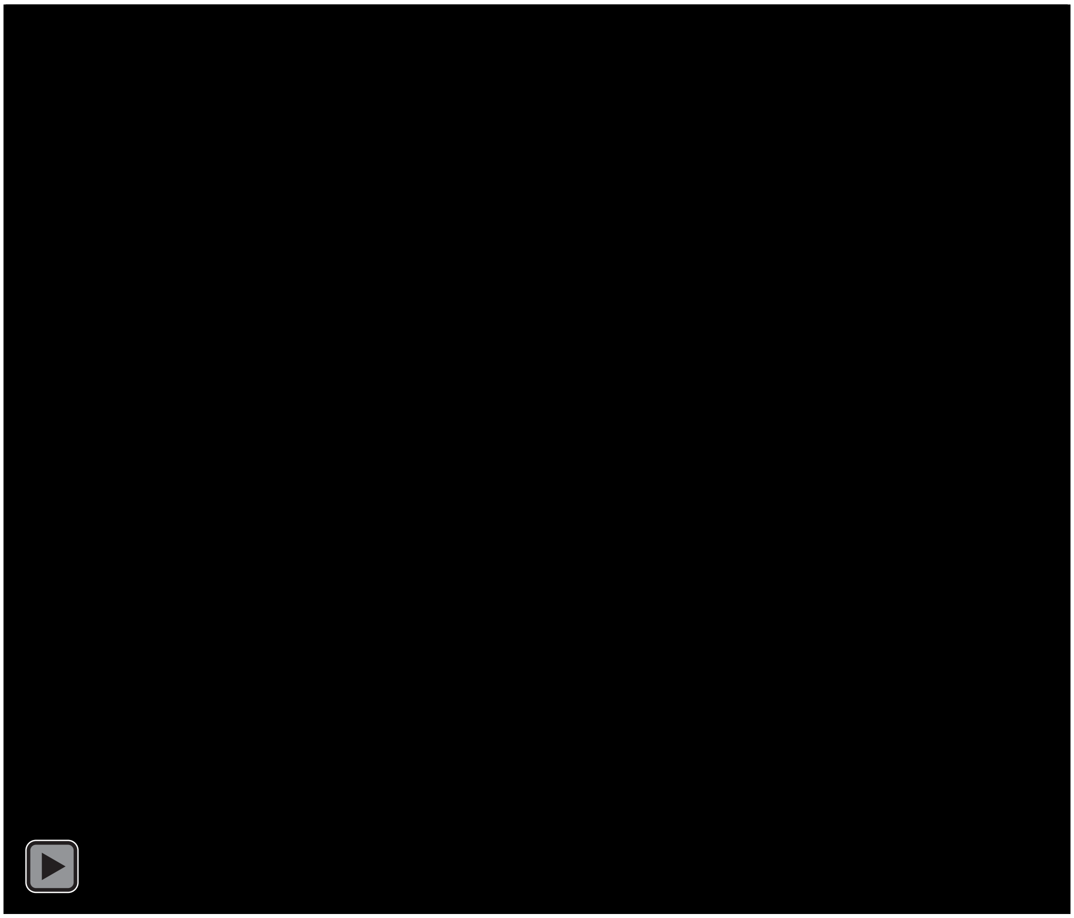

Fig. S1. Fluctuating SASE pulses focused by the Be-CRL stack. The color bar adapts for each frame. The scale bar indicates 1 mm.

IUCr macros version 2.1.11: 2019/01/14

3. Evaluation of Pre-Processing and its Effect on the Reconstruction

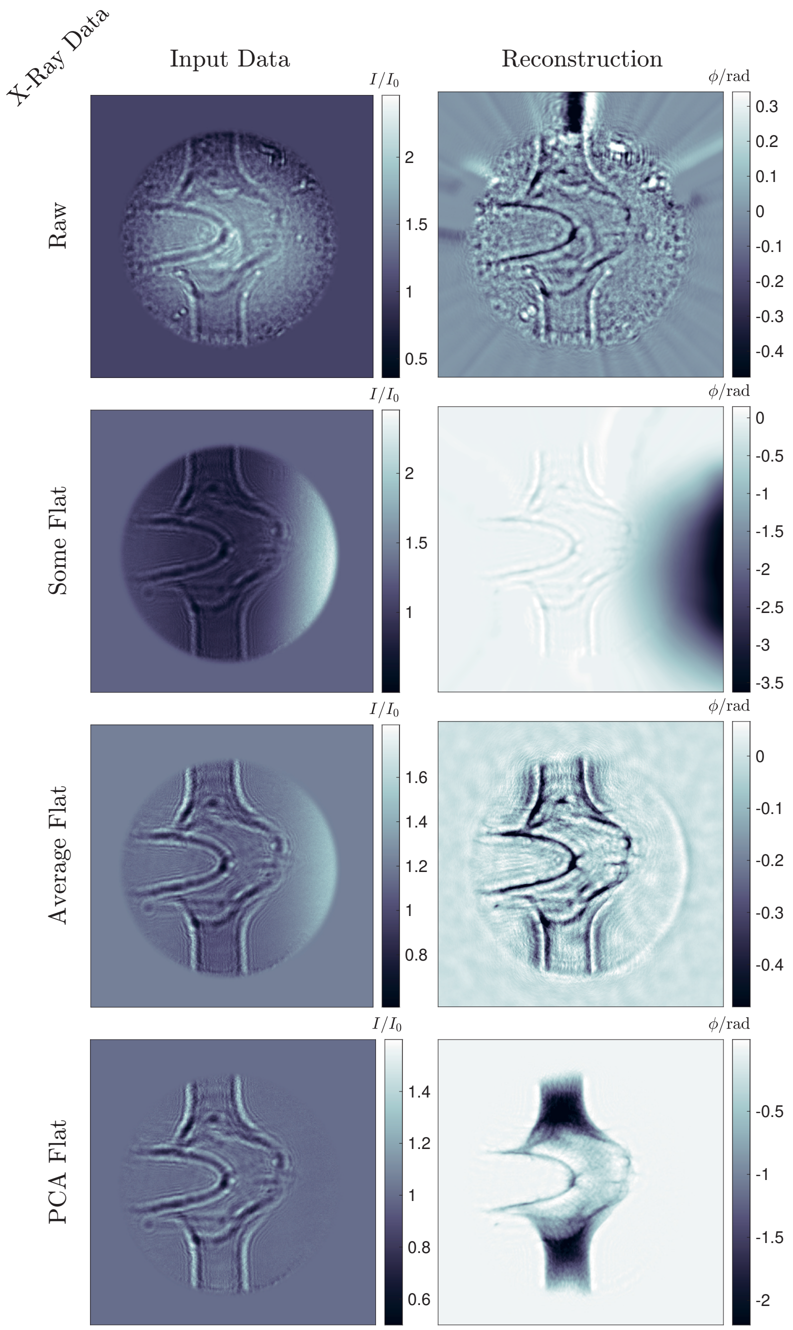

Fig. S2. Illustration of the effect of the chosen pre-processing on the final reconstruction result. From top to bottom the pre-processing scheme gets more advanced, starting with a raw hologram (dark current corrected and constant padding added) and then following flat-field correction by (i) an arbitrary empty beam image, (ii) an average empty image and (iii) the flat-field synthesized from the PCA.

In order to achieve optimal results from the phase reconstruction, the input data (the flat-field corrected hologram) should satisfy certain criteria:

(i) the I_0 has to be matched to avoid global offsets

(ii) intensity gradients should be avoided

(iii) the corrected image fades to 1 on the boundary

(iv) small structures stemming dust particles or the like are not present anymore.

Figure S2 illustrates the effect of the pre-processing on the obtained reconstruction. The left column shows the input data for the algorithm explained in the main text. The right column shows the obtained reconstruction from the input.

In order to obtain a reconstruction from the raw data, the intensity has to to vary in a value range around 1. This corresponds to the (in this case not satisfied) assumption of an illumination with a plane undisturbed wave. Thus an area in the lower right of the illuminated area has been chosen to determine an average intensity. This value has been used for normalization.

The next case uses 'some' empty image to carry out the flat-field correction. In this case multiple outcomes are possible e.g. if the overall intensity of the empty pulse and the measurement do not match or if the empty image has a strong intensity gradient (caused by a different pointing), which is the case shown in the figure. These effects can be moderated to some extend by correlation approaches but one will be always limited to chose from the set of empty pulses which have been measured.

The third row depicts the flat-field correction by an average empty pulse image obtained from averaging over 30 images. Compared to the previous variant we obtain for this particular measurement a better correction.

Finally we carry out the flat-filed correction with a synthesized empty beam image obtained from PCA. This image fulfills all the quality indicators listed above and is the optimal input for the phase reconstruction, as we discuss in the following.

Essentially the phase reconstruction decodes phase information from intensity information. This means the algorithms tries to attribute some object information to each measured feature in the intensities. In the reconstruction from the raw hologram we notice, that the algorithm reconstructs all small dirt particles stemming from the Be-CRL, also the conturs of the jet are recovered but neither the details nor the overall phase shift is properly recovered. The phase range seems to shallow.

The next reconstruction obtained from the 'Some Flat'-correction shows a large gradient disturbing the image. The Be dirt is not present any more, but also in this case only the contours of the jet are recovered. Filtering operation could remove the gradient, but would not 'fill up' the missing low spatial frequency information in jet.

The quality of the 'average flat' reconstruction compared to the previous one is again improved. Here the overall background is flat, but the transition from the measurement to the padding is pronounced. Features are retrieved well, but not large phase shifts.

The reconstruction obtained from the PCA computed flat-field shows both small features and correctly recovered large phase shifts. Overall this case yields the most faithful recovery. The correct recovery of low spatial frequencies becomes of interest if different materials should be distinguished.

The reconstructions were obtained with 1000 iterations of AP. Note, that the better the pre-processing of the input data is, the more strict the applied constraints can be e.g. the automatic support determination did not work in the first 3 cases since the stray structures besides the actual object can not be excluded. Also the range constraints needs to be relaxed for the first 3 cases in order to obtain the images shown here. This meant allowing $\bar{\delta} > 0$ and $\bar{\beta} \in [-0.2, 0.2]$, applying the constant ratio $\bar{\delta}/\bar{\beta}$ did not yield usable results.

4. Number of Components

Figure S3 illustrates the flat-field correction for an increasing number of components used for computing the corresponding flat-field. For $N_C \leq 10$ we observe mainly low spatial frequency shifts of the background. The low spatial frequencies are well described for $N_C \approx 10$. The influence of higher components is illustrated by the zoom on the ripple-like artifact, see Fig. S4. The ripple artifact stems likely from a dust particle on the nano-focusing CRL. In the full field of view (FOV) it is located in the lower third on the axis of the jet. In the zoom movie a different behavior compared to the full FOV is visible. For $N_C \leq 10$ the changes of the artifact's structure are only minor. For $10 \leq N_C \leq 25$ the effect of the higher components becomes visible as jumping of the structure and (desired) decrease in contrast of the artifact. Ideally the artifact would completely disappear, but this is not the case due to limitations inherent in the standard flat-field correction based on dividing intensities (Homann *et al.*, 2015).

Figure S5 shows all 50 components obtained by the PCA. The inspection of the higher components shows only faint structures. A notable exception is again the ripple artifact which appears again in different versions. The ripple's jumping behavior is also observable in the empty beam movie, see Fig. S1.

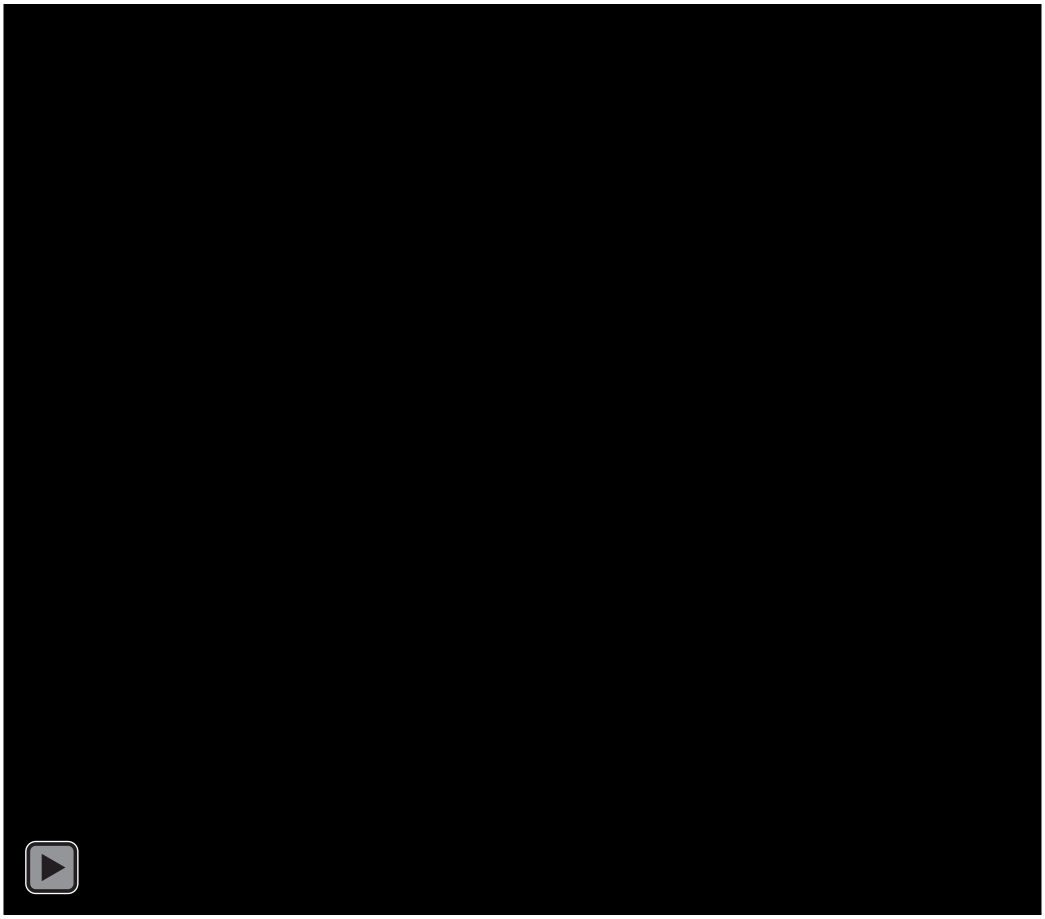

Fig. S3. Flat-field correction with increasing number of components used for computing the linear combination on a particular hologram. Not all divisions are shown, from $N_C = 10 - 20$ every second and from $N_C = 20 - 50$ every fifth division result is shown.

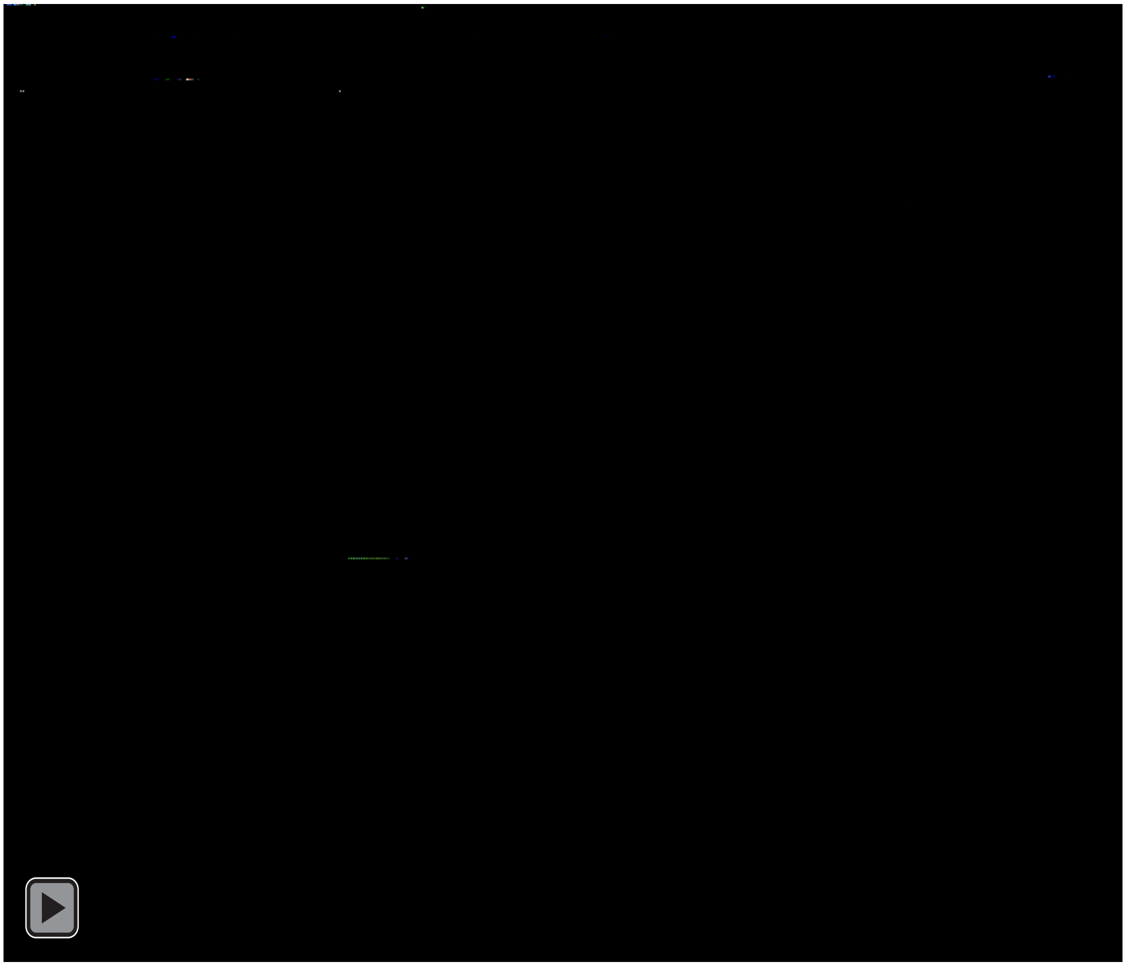

Fig. S4. Zoom on the ripple-like artifact. Same frames shown as in Fig S3.

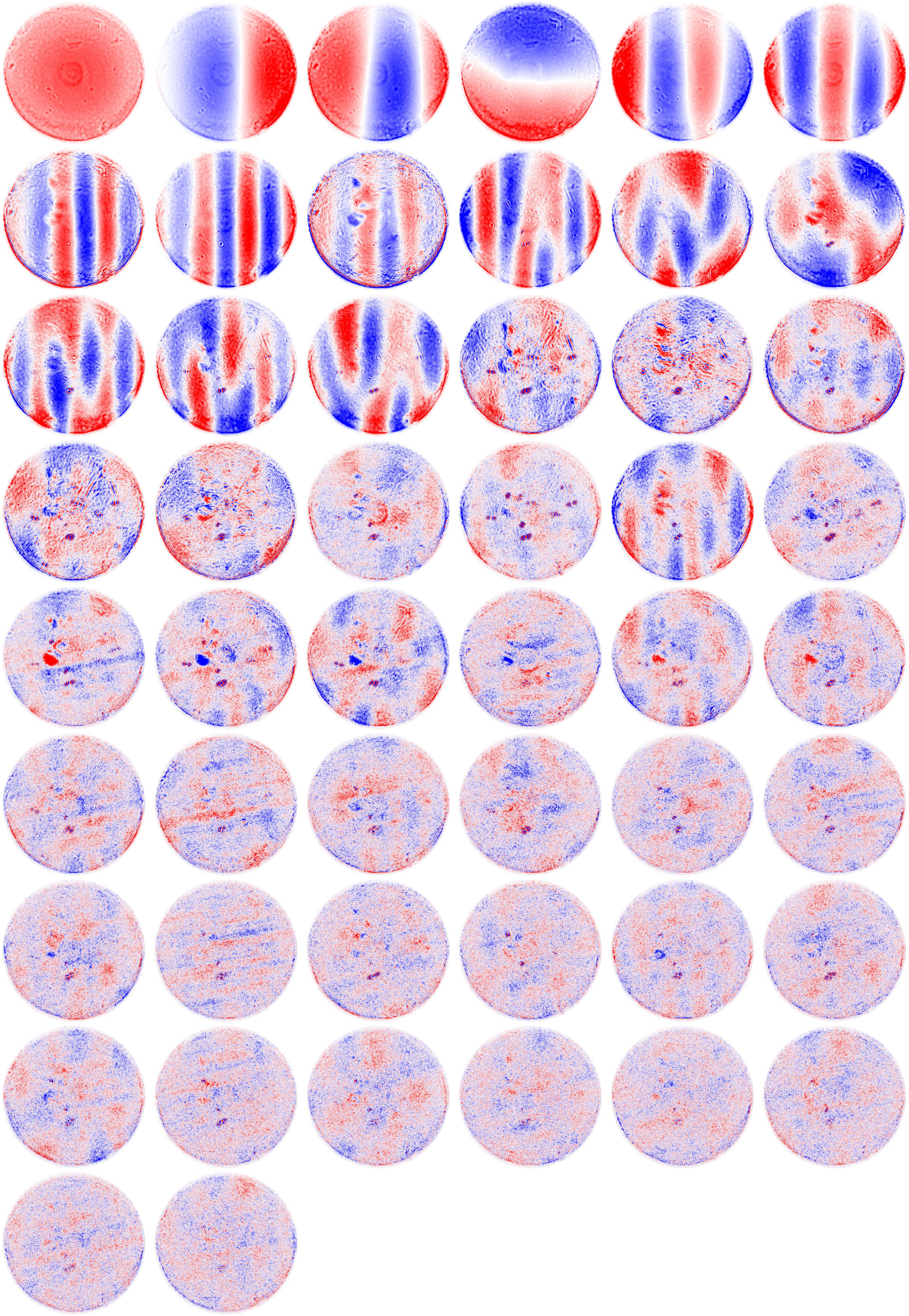

Fig. S5. All 50 componets extracted by the PCA for run 246. Same color coding as in the main text.

5. Jet Movie

Figure S6 shows a movie of the intrinsic dynamics of the jet, before the break-up. Figure S7 shows the animated version of Fig. 8 from the main text.

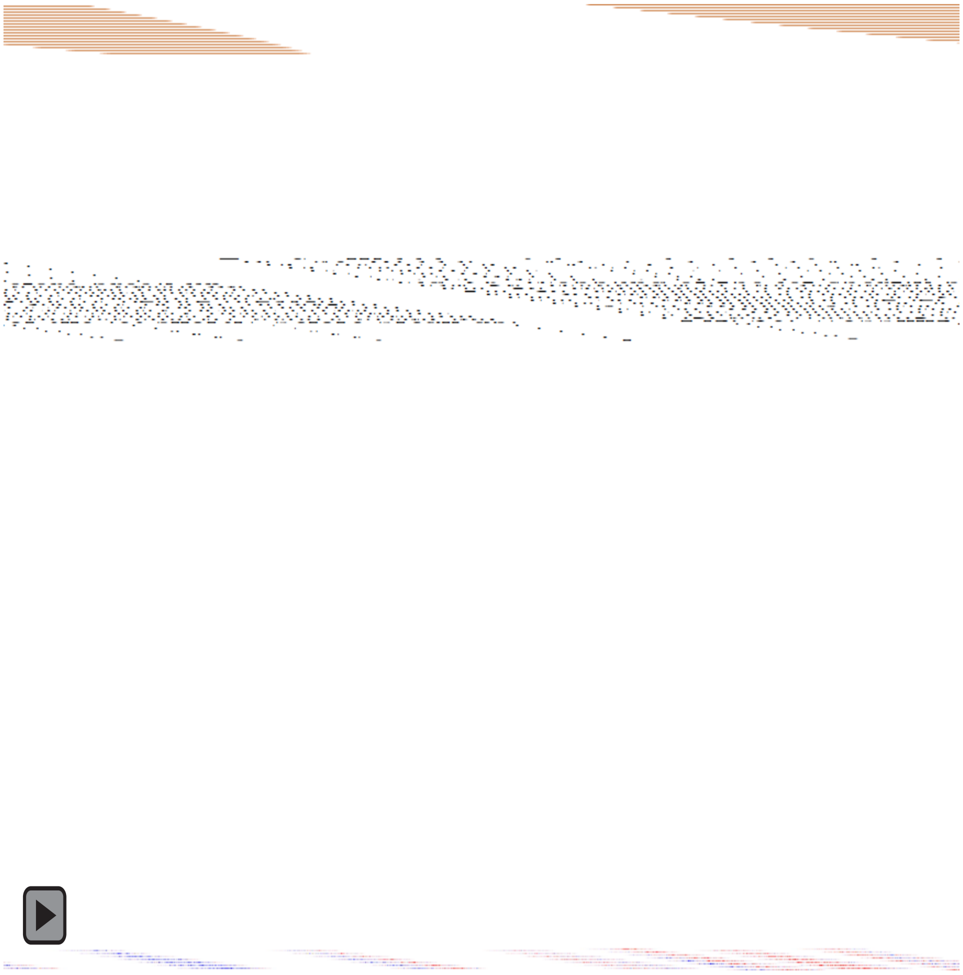

Fig. S6. Wobbling of the jet shortly before break-up.

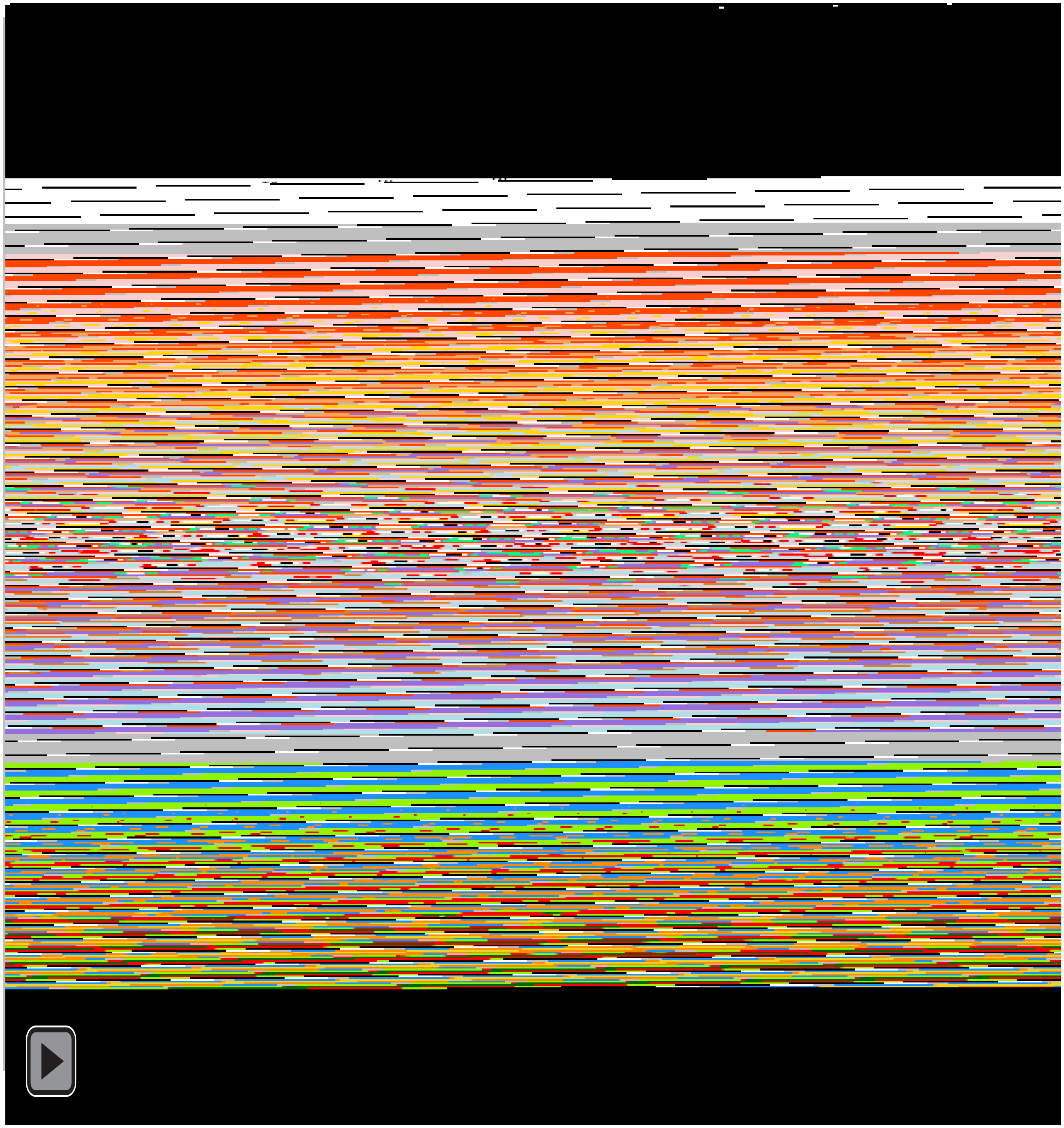

Fig. S7. Animated version of Fig. 8 of the main text.

6. In-air Shock Wave

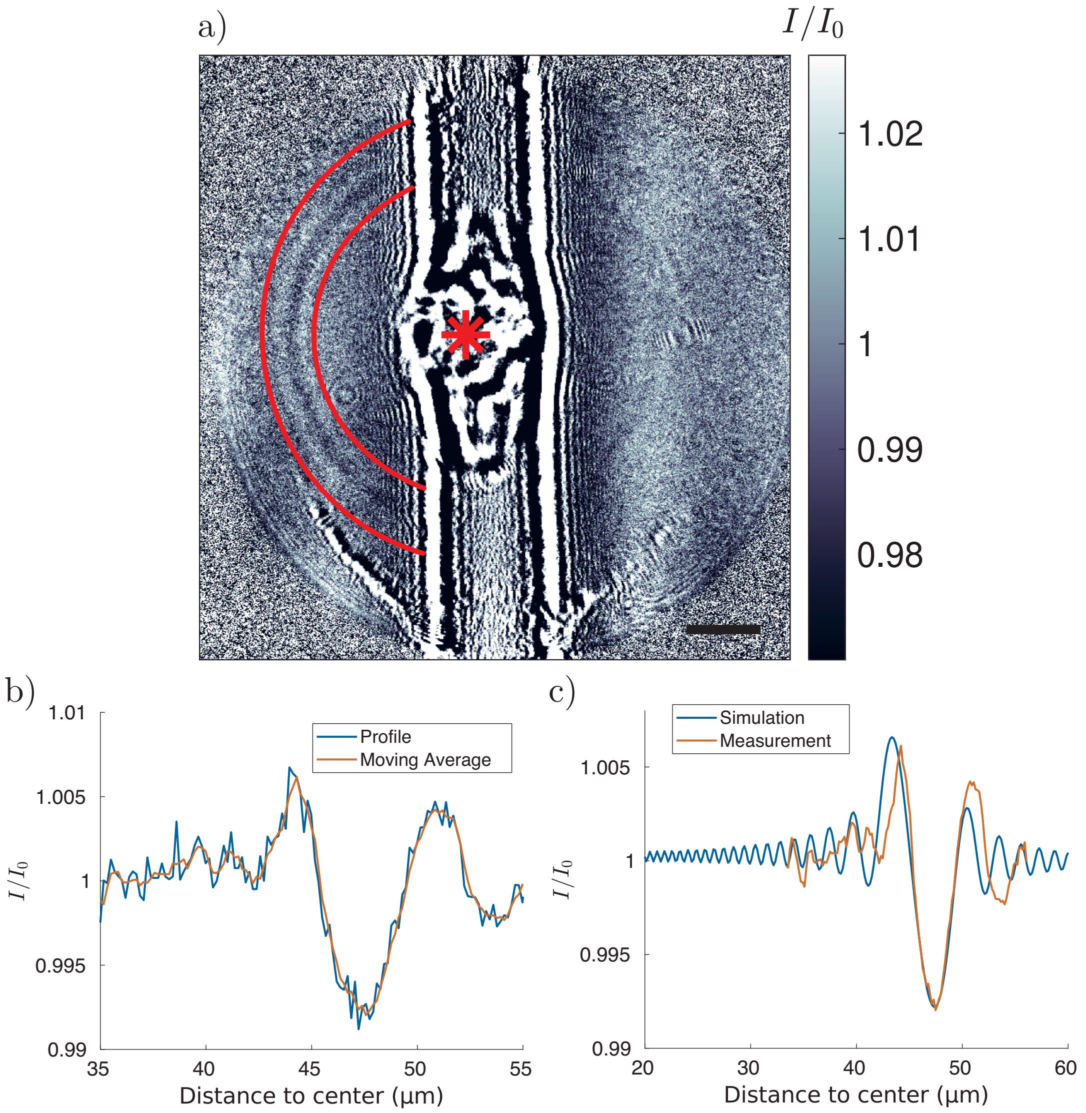

Fig. S8. Shock wave in air recorded with single-pulse illumination. (a) shows the flat-field corrected hologram of the exploding jet 6 ns after the IR-laser pulse. The fringes of the in-air shock-wave are framed by the two red lines. The center of the shock wave is indicated by the asterisk. (b) The extracted intensity profile from the region marked red in (a). (c) By forward modeling the radius of the shock wave was estimated to be 46 µm.

For x-ray pulse delays shorter than $\Delta t < 9\,\mathrm{ns}$ the shock wave in air is visible as well. Figure S8(a) shows a hologram of the jet 6 ns after pumping with the IR laser. The contrast in the image had to be increased considerably in order to make the in-air

shock wave visible. The red arcs indicate the shock wave on the left. At the same time this is the region which was used to extract an intensity profile of the shock wave shown in (b). Small background variations already disturb the visibility of the shock wave. As can be seen on the right-side of the water jet.

Given the field of view(FOV), the placement of the jet in the FOV and the visibility issues, the in-air shock waves are only visible in a short range of time delays shorter than 9 ns. These conditions complicate the analysis of this phenomenon. The following analysis has been carried out exemplary on the hologram shown in Fig. S8(a). First, the center of the shock front had to be determined. For this we have used the masked shock wave and calculated the angular average for systematically varied centers of the shock wave. These obtained intensity profiles were smoothed by a 4 pixel moving average, then the profile with the highest contrast, according to $(I_{\max} - I_{\min})/(I_{\max} + I_{\min})$ has been selected, see Fig. S8(b). The highest contrast was found to be 0.7%. The corresponding center has been marked by an asterisk in the hologram.

Secondly, the radius of the shock wave was determined. This has been achieved by forward simulations of a projected phase sphere of varied phase shift δ_{shock} and radius r_{shock}. The holograms of these spheres have been propagated using a Fresnel number of $\text{Fr} = 1.71 \cdot 10^{-3}$ (same values and definitions apply as in the main text) and then angularly averaged. The best fitting profile is shown in Fig. S8(c). The profile corresponds to parameter values of $r_{\text{shock}} = 46\,\mu\text{m}$ and $\delta_{\text{shock}} = 8.41 \cdot 10^{-9}$. Using the time delay and radius a shock velocity of $v = 7.6\,\text{km/s}$ is estimated.

7. Data Availability

The data (Hagemann *et al.*, 2019) of the experiment will be available after **2022-10-14** through EuXFEL online services.

References

Hagemann, J., Scholz, M. & Salditt, T., (2019). Cavitation dynamics studied by time-resolved high-resolution x-ray holography (p2544, derived from p2207). DOI: 10.22003/XFEL.EU-DATA-002544-00.

Homann, C., Hohage, T., Hagemann, J., Robisch, A.-L. & Salditt, T. (2015). *Phys. Rev. A*, **91**, 013821.
